# Recipes

**Published:** 1874-02

**Authors:** 


### RECIPES.

### BAKING MEATS.

In roasting or baking meats or poultry no water should be used; it greatly impairs their flavor. Instead of water, put a little lard or dripping in the pan. Have the oven thoroughly hot when the meats are put in, so that a crisp may be formed at once, which will retain the juices. If the meat is likely to burn, lay over it a piece of thin greased paper.

### COTTAGE PUDDING.

One cup of butter, 2 cups sugar, 1½ cups sweet milk, 3½ cups flour, 1 teaspoonful soda and 2 of cream tartar. Flavor with vanilla.

### FRUIT PUDDING.

One teacupful of beef suet chopped fine, 1 teacupful of molasses, 1 do. of sweet milk, ½ pound raisins chopped, 1 teaspoonful soda (no cream tartar), 1 teaspoonful salt, 1 do. ground cinnamon, 1 do. cloves, ½ a nutmeg, 3 cups flour. Boil 4 hours in a tin pudding mould. The water must be boiling when put in and kept boiling until the pudding is taken out.

### SAUCE FOR ABOVE PUDDINGS.

One teacupful of butter, 2 do. pulverized sugar, beaten to a cream in an earthen dish. Then add a little less than a cup of sherry wine, stirring it in a teaspoonful at a time until the mass is blended into a light cream. Ten minutes before using put the dish in a pan of boiling water on the stove, stirring the sauce till quite hot, but not boiling.

